# Design and performance analysis of salinity gradient solar pond under different climatic and soil conditions

**DOI:** 10.1371/journal.pone.0279311

**Published:** 2023-02-09

**Authors:** Muhammad Ihsan Shahid, Muhammad Asim, Muhammad Farhan, Muhammad Fahad Sheikh, Muhammad Usman Ashraf, Hassan Arshad, Ahmed Alghamdi, Abdullah S. Alshahrani, Adel A. Bahaddad, Khalid Ali Almarhabi

**Affiliations:** 1 Department of Mechanical Engineering, University of Management and Technology, Sialkot Campus, Sialkot, Lahore, Pakistan; 2 Department of Mechanical Engineering, University of Engineering and Technology, Lahore, Pakistan; 3 Department of Computer Science, GC Women University Sialkot, Sialkot, Pakistan; 4 Department of Software Engineering, College of Computer Science and Engineering, University of Jeddah, Jeddah, Saudi Arabia; 5 Department of Computer Science and Artificial Intelligence, College of Computer Science and Engineering, University of Jeddah, Jeddah, Saudi Arabia; 6 Department of Information System, Faculty of Computing and Information Technology, King Abdulaziz University, Jeddah, Saudi Arabia; 7 Department of Computer Science, College of Computing in Al-Qunfudah, Umm Al-Qura University, Makkah, Saudi Arabia; Birla Institute of Technology and Science - Hyderabad Campus, INDIA

## Abstract

A salinity gradient solar pond (SGSP) is capable of storing a significant quantity of heat for an extended period of time. It is a great option for providing hot water at a reduced energy cost. Additionally, SGSP is used in low-temperature industrial applications such as saltwater desalination, space heating, and power generation. Solar pond thermal performance is dependent on a variety of operational variables, including the soil conditions, the climate of the particular site, the thickness of the solar pond layers, the depth of the water table, and the salt content of the pond. As such, this study examines the thermal performance of a solar pond under a variety of operational conditions. The solar pond model is used to test the thermal performance by simulating two-dimensional heat and mass transport equations. The equations are solved using the finite difference technique utilizing MATLAB® scripts. Salt distributions and temperature profiles are computed for a variety of factors influencing SGSP’s thermal performance. The main distinguishing variables influencing the thermal performance of SGSP are soil conditions, such as soil texture, types, the moisture level in soil, and water table depth. The final findings indicated that the fine sand dry soil performed better than the other soil types owing to its poor heat conductivity. The economic results indicated that the period of return (POR) of the intended system is around 2 years. The solar pond construction costs such as excavation, transportation, salt and lining, were considered based on the local prices. This modeled study extracted the greatest possible energy is 110W/m^2^, with the fine sand dry at 62.48°C lowest temperature. This study suggested that the climatic conditions of Lahore is better than climatic conditions of Islamabad. Additionally, deeper water tables are suggested for improved thermal performance of the pond.

## 1 Introduction

Renewable energy sources have become more important in recent years. SGSPs are low-cost solar collectors for long-term storage that can absorb solar energy as heat and distribute it continuously throughout the day and night [1, 2]. Due to the effect of high-intensity solar radiation and the cheap cost of brine, SGSP may be a very attractive option for heat collection and storage. Additionally, solar ponds may contribute significantly to the growing need for pollution-free energy by providing electricity at a competitive price [3, 4]. In contrast to the flat plate collector, the SGSP gathers less heat during bright days owing to its lower effective absorptivity [5] but absorbs more heat during overcast days due to its lesser loss factor. Because of their large heat storage capacity and low daytime temperature variation, SGSPs have a variety of applications, including generating electricity [6], desalination [7, 8], building temperature control, and industrial operations heating [9, 10].

**Fig 1:**
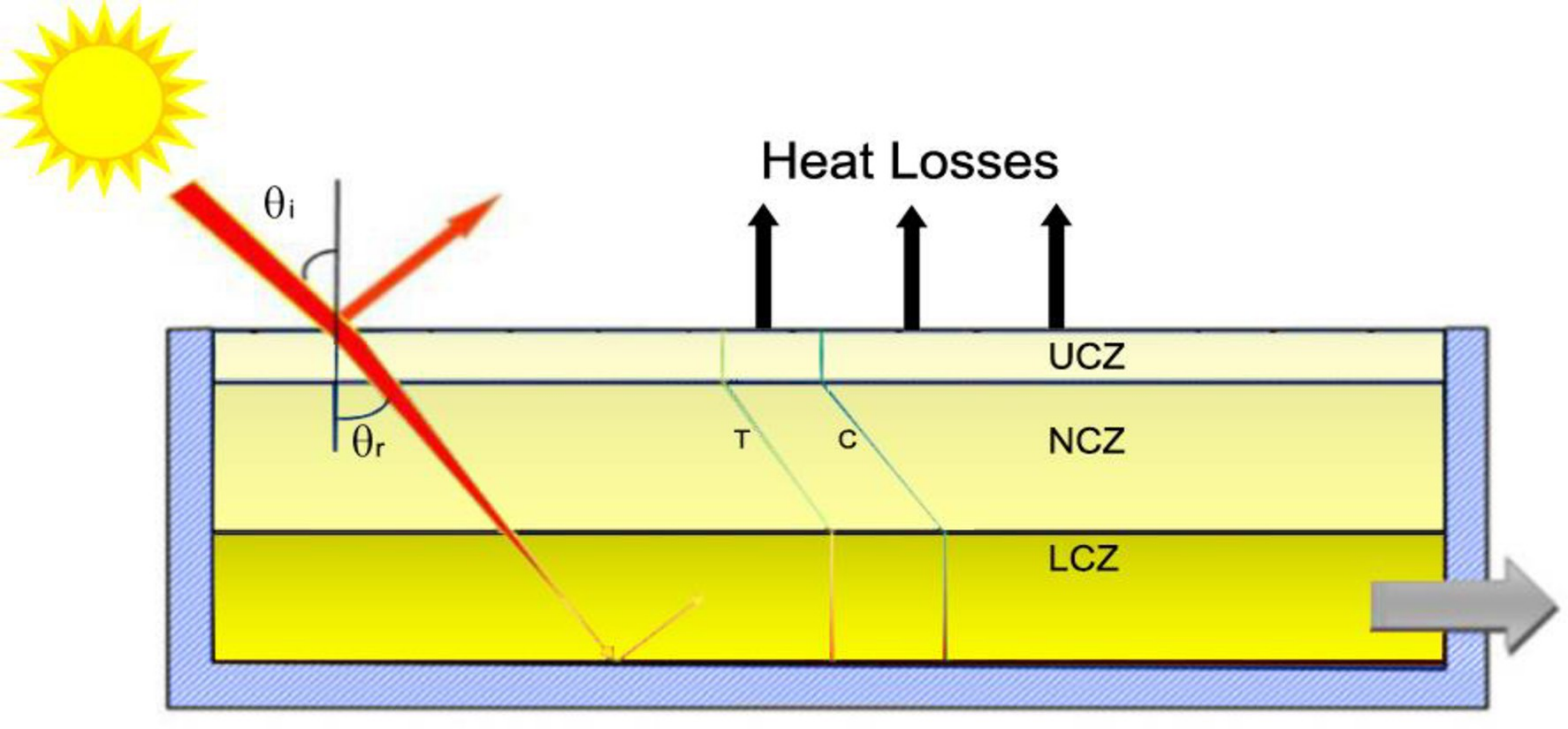
Schematic view of SGSP.

Fig 1 depicts the overall design of a three-zone solar pond. The Upper Convective Zone (UCZ) includes fresh water at ambient pressure and has a low salt content and density due to its thickness ranging between 0.2 and 0.4m. The Non-Convective Zone (NCZ) acts as a transparent insulator in the pond, preventing convective heat loss. NCZ has a progressively changing salt density as the depth of this specific zone increases, with a thickness ranging from 0.5 to 1.0m. Because of its high salt concentration and density, the top layer, known as the Lower Convective Zone (LCZ), retains heat. The pond’s bottom surface is painted with a black surface. The sun’s rays penetrate through the top layer of water and hit the black-painted pond’s bottom, absorbing them. The heated water does not flow higher due to the high density of the solution, resulting in a heat storage zone [11, 12].

Researchers have been attempting to improve the overall efficiency of solar pond systems in recent years [13, 14]. The permanence of the salinity gradient is essential for the solar pond technology to work properly [15]. Experimental investigations in prototype or industrial solar ponds are difficult to conduct, and most published studies use theoretical models [3, 16].

Assad H. Sayer et al. [17] developed an SGSP model and a MATLAB program that utilized the Runge–Kutta numerical technique to solve first-order differential non-linear equations. Ali et al. [18] performed experimental assessment to examine the thermal performance of the mini PV/solar pond for which, system’s highest overall efficiency value was 37.67 percent in September. Additionally, September had the greatest daily thermal efficiency value of 30%, while December saw the highest daily electrical efficiency value of 9.357%. Rghif et al. [19] conducted a numerical study of the Dufour and Soret effects on heat and mass transfer inside an SGSP and its storing efficiency in the Tangier (Morocco) climatic circumstances. The impacts of Soret and Dufour on thermosolutal convection are investigated numerically in this research using an SGSP. In this study Heat and Mass Transfers were described by the Navier-Stokes, thermal energy and diffusion equations which were solved with a finite volume scheme and the Gauss method.

Kumar et al. [20] presented a novel transient-state analytical model to forecast the performance. Proposed methodology was based on the expansion of Eigenfunctions to predict temperature profiles within the solar pond having dual layer heat extraction. Hamdeh et al. [21] presented a redesigned spiral piping network and conducted an extensive computational analysis. The involved governing equations were calculated by using the Finite Volume Method (FVM) through sequential repetitive calculations. The 3D model of spiral pipe was designed by CATIA V.5R21 and simulated by ANSYS Fluent 15.

Cao et al. [22] presented a system comprised of a Kalina cycle (KC) and Two Thermoelectric Generators (TEGs) combined with a Humidification-Dehumidification Unit (HDH). A parametric study is carried out and the effects of important design factors on the system performance and cost metrics were investigated. EES software to model the system mathematically. Chakrabarty et al. [23] conducted a transient study to determine the impact of different factors on the temperature change of a solar pond, including ground conditions, zone thickness change, and surface losses using finite difference method.

Kumar et al. [24] investigated the effect of side insulation in an SGSP and the trade-offs between different operational parameters in steady and unsteady conditions. While the steady-state model is computed analytically, the unsteady-state equations are solved numerically employing an intuitive finite difference method. Iman et al. [25] investigated the effect of layers, as well as the different climate conditions, on the temperature in the ponds bottom layer. Several analyzes were performed for this purpose employing the Taguchi method. Assari et al. [26] determine the effects of the PCM on the stability of the solar ponds, its operating principle, stability criteria and methods, salinity gradient formation, obstruction, and remedies. Colarossi et al. [27] analyzed the stability of pond through a laser shadowgraph technique, to visualize the effect of the thermal convection on the interfaces.

Mosaffa et al. [28] studied organic Rankine cycle power production system might be integrated with a salinity gradient solar pond. A numerical model of SGSP is developed in MATLAB Software using a finite difference method. In this case, the system generated power is estimated of 95.67 MJ year^-1^ m^-2^ with an energy efficiency of 3.28%. Rghif et al. [29] conducted a numerical investigation on the Dufour effect and the effects of a layer of Phase Change Material (PCM) on the thermal efficiency of an SGSP. Heat and Mass Transfers are described by the Navier-Stokes, thermal energy and diffusion equations which are solved with a finite volume scheme and the Gauss method. Tian et al. [30], In this study, an active method of using an external magnetic field is proposed to repress the intense convection region and improve its corresponding operating stability. A two-dimensional transient model is developed and solved using the lattice Boltzmann method with multiple-relaxation-time collisions. Yassmine et al. [31] developed the numerical model in Fortran 95 based on 2D Navier-Stokes, thermal energy and diffusion equations with an appropriate treatment of the SGSP boundary conditions. These equations are solved using the implicit Finite Volume Method and the SIMPLE algorithm. Numerous methods have been suggested in the literature for numerical calculation of the governing solar pond’s partial differential energy equations. Safwan Kanan et al. [32] simulated the system performance in Baghdad, Iraq, using a transient SGSP model in MATLAB. A finite-difference approach was utilized in [19, 33, 34], whereas a finite element methodology was used in [20, 35] to control the solar pond. Numerous numerical [1, 22, 36] and analytical models [2, 21, 37] for describing and forecasting the temperature profiles in SGSPs have been presented. Other recent experimental investigations on solar ponds [9, 23, 24, 27, 38, 39] have been conducted to examine and measure the solar pond’s performance in various ways. Furthermore, a loss through the surface underneath the SGSP significantly impacts its thermal performance. Losses horizontally from the sidewalls are measured and estimated even though the model has a high degree of agreement with actual data [40].

The lack of research in literature is that there is no study available to analyze the performance of solar pond under climatic conditions of Pakistan for different soil conditions. Novelty of this research lies specifically in the case study of solar pond under the different climatic and soil conditions of Pakistan and to evaluate the potential of this technology in this region of the world. In this study, simulation is done by using finite difference technique under climatic conditions of Lahore, Islamabad and results have also validated with an experimental study. Also conducted the thermo-economic analysis to examine the short payback period. Solar pond stored the 963 kwh/m^2^ annual yield and control the 4336.2 kg emissions of CO_2_ per annum for other heating purposes. Moreover results represent the Lahore location, because there is great potential for the construction of solar pond in this area.

## 2 Proposed methodology

Pakistan’s climate changes throughout the year owing to seasonal variations, and Pakistan is divided into several areas based on climatic variables [41]. As a result, the areas of Pakistan chosen have varying climates. The proposed study examines the effect of climatic variables in various places across Pakistan, soil conditions (texture, type, and moisture content), water table depth, and layer thicknesses on the thermal efficiency of SGSP. Two cities in Pakistan were chosen for the research of SGSP: Islamabad (33.6844° N, 73.0479° E) and Lahore (31.5204° N, 74.3587°). The proposed model used SGSPs with a surface area of 1m^2^, a depth of 0.3m for UCZ, 1m for NCZ, and 0.7m for LCZ.

**Fig 2:**
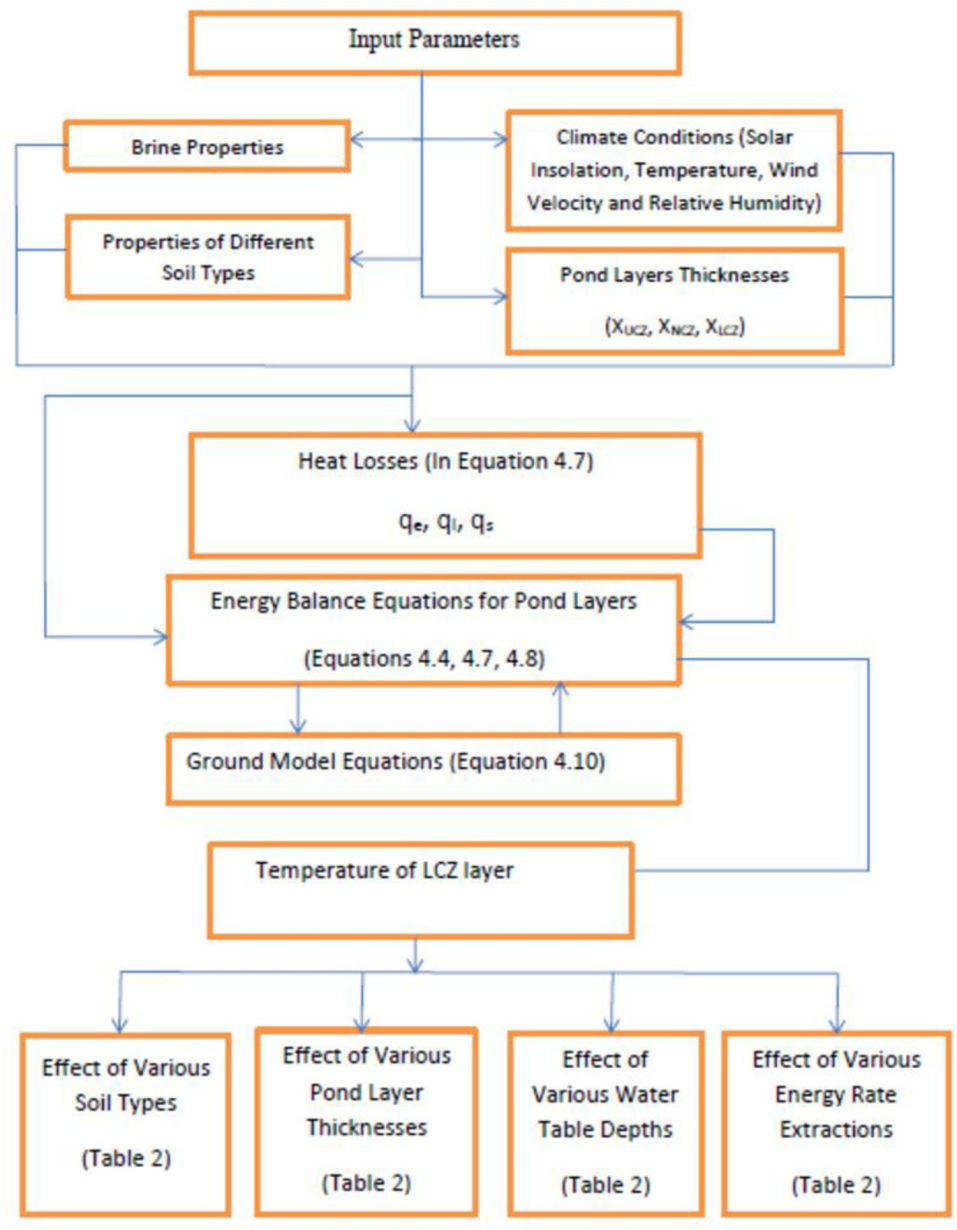
Flow chart showing solution procedure on MATLAB.

SGSP may be thought of as a two-dimensional model with an internal heat source. The differential equations in this model are solved using the finite difference technique in MATLAB software as the flow chart of program depicts in Fig 2. This technique was utilized to characterize thermal movements inside the SGSP and under the pond. The SGSP was split into three horizontal layers: UCZ, which has a constant temperature, and LCZ, which has a variable temperature and is regarded as a convective zone. The ground model and NCZ are subdivided further into sub levels.

### 2.1 Data collection

#### 2.1.1 Soil thermal properties

Heat loss from a solar pond is influenced by the thermal characteristics of the soil underneath it [42]. The amount of heat lost via conduction from the pond to the ground is dependent on the thermal conductivity, specific heat, and density of the soil [43, 44]. The thermal conductivity (*k*g) was measured from the given equations by [29] as follows:

$$k_{g}=0.14423(0.9\;\log\;\omega -0.2)\;10^{0.000624_{pgd}}\;for\;clay\;and\;silt\;soils$$ (2.1A)

$$k_{g}=0.14423(0.7\;\log\;\omega +0.4)\;10^{0.000624_{pgd}}\;for\;sand\;soils$$ (2.2B)

For Lahore and Islamabad, the soil thermal characteristics utilized in this study are listed in Table 1.

**Table 1:** Soil thermal properties [29, 32, 43, 44].

Soil type	Thermal	Density (ρ)	Specific Heat (C_p_)
	Conductivity (K_s_)	(K_g_/m^3^)	(KJ/kg.°C)
	(W/m.°C)		
Sand soil	1.082	1352	1105
Clay and silt soil	2.15	1350	1137
Clay and silt soil	1.9322	2325.25	1007.34
(5% moisture content)			
Clay and silt soil	3.152	2255.5	1175
(10% moisture content)			
Coarse sand (dry)	0.25	1800	800
Fine sand (dry)	0.15	1600	800

#### 2.1.2 Weather data

**Fig 3:**
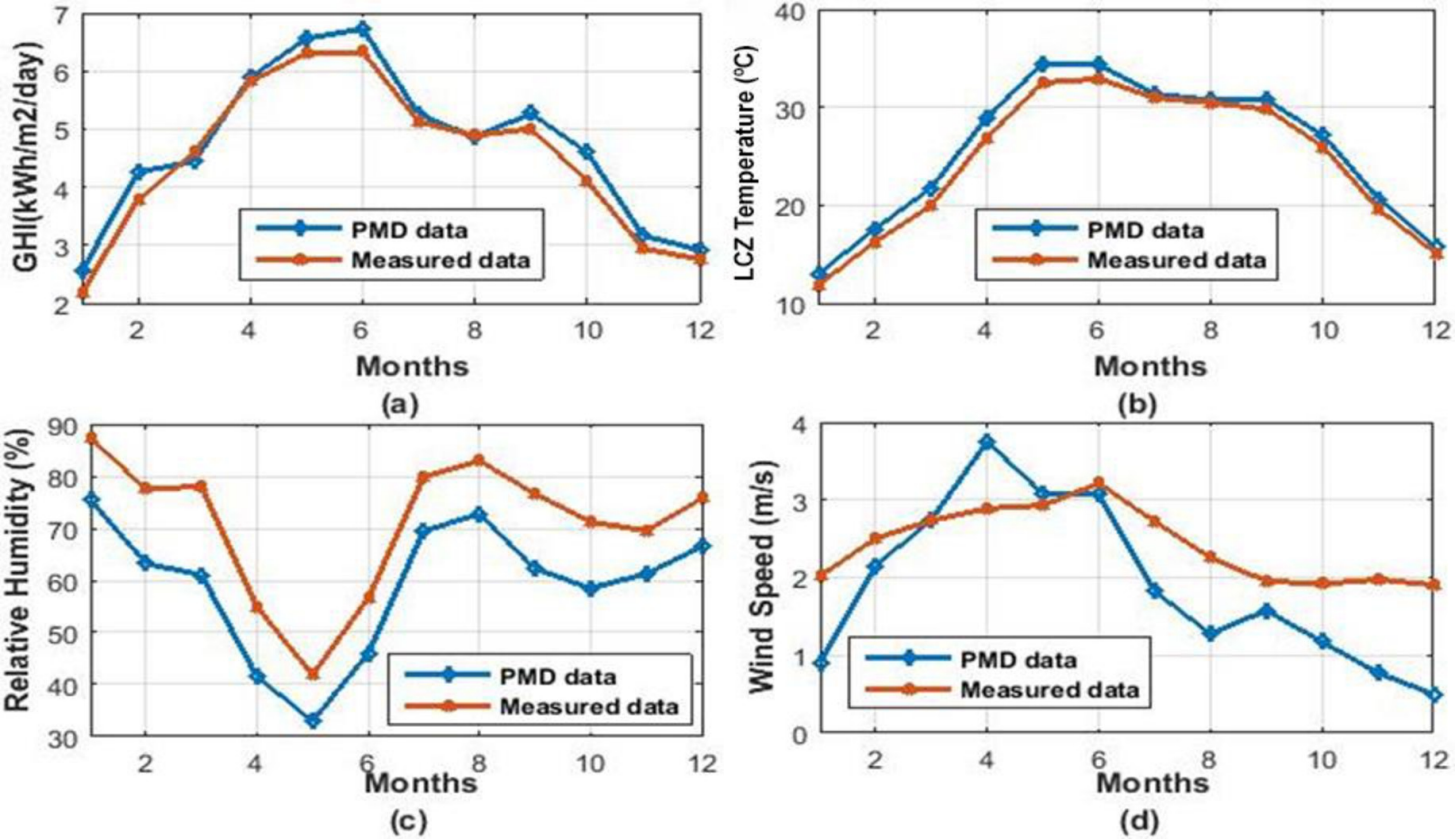
Weather data of Lahore. **(a)** Global Horizontal Irradiations (kWh/m^2^/day) **(b)** Dry bulb Temperature (Celsius) **(c)** Relative Humidity (%) (**d)** Wind Speed (m/s).

**Fig 4:**
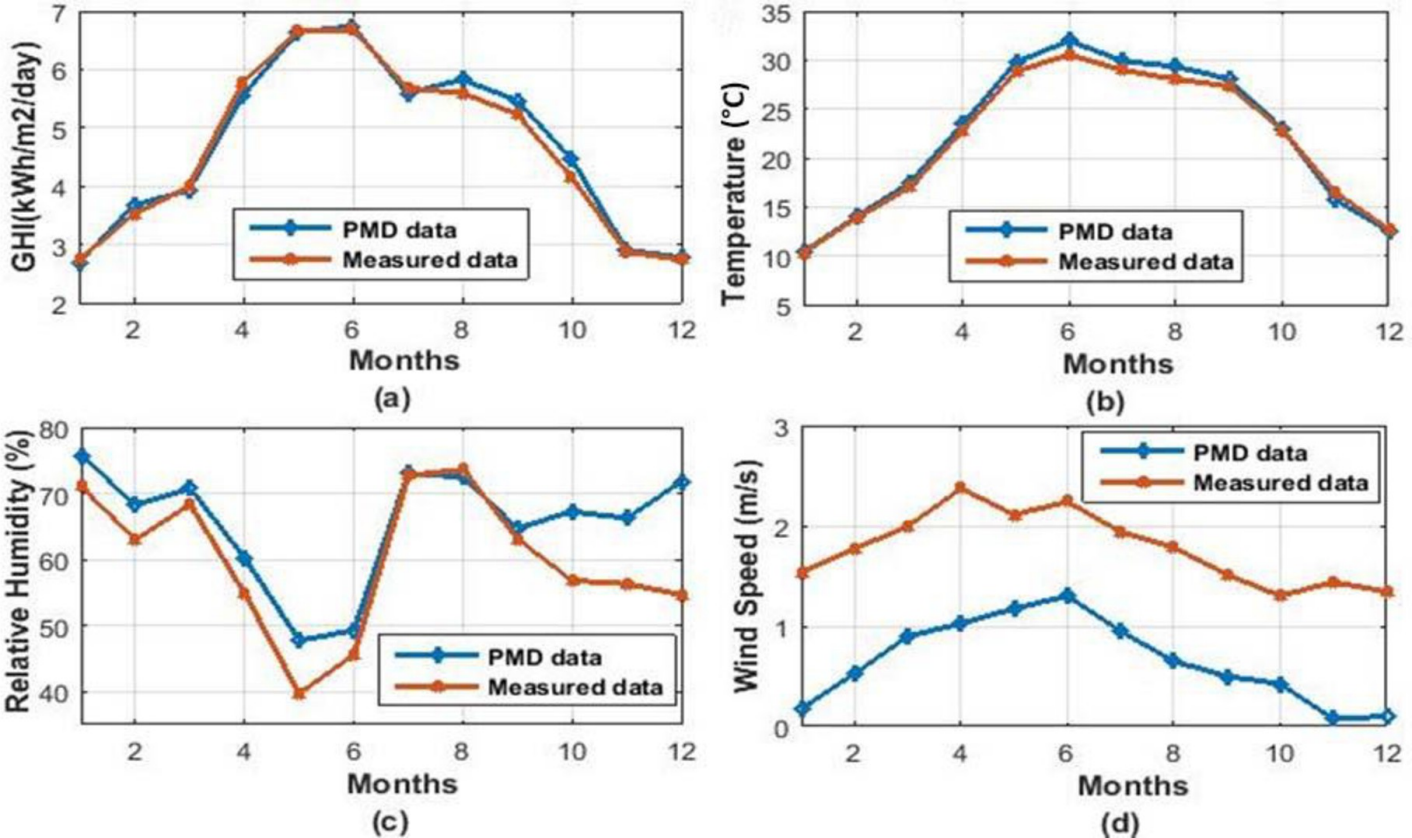
Weather data of Islamabad. **(a)** Global Horizontal Irradiations (kWh/m^2^/day) **(b)** Dry bulb Temperature (Celsius) **(c)** Relative Humidity (%) (**d)** Wind Speed (m/s).

Temperature, Global Solar Irradiation, Wind Speed, and Relative Humidity are the critical meteorological variables that influence the functioning of a solar pond. In this research, the average monthly data (2014–2019) for various cities in Pakistan was obtained from the Pakistan metrological department, Lahore [44], and supplemented by the measured data (2014–2019) from UET Lahore [45]. Figs 3 and 4 demonstrated that this study utilized both measured and PMD recorded data (2018–2019) for Islamabad and Lahore, two Pakistani metropolitan cities.

### 2.2 Model development and analysis

#### 2.2.1 SGSP and ground model

2.2.1.1 Following assumptions were made to solve the pond’s heat transfer equations and replicate the mathematical pond model [45–47].

- The SGSP is divided into three different zones: the UCZ, the NCZ, and the LCZ.
- In UCZ and LCZ, the temperature gradients and density are uniform.
- The heat losses from the ground underneath and pond walls are monitored and calculated.
- To enhance solar radiation absorption by darkening the bottom surface.
- As a result, the energy emitted by the sun that reaches the Lower region is completely absorbed by the salt solution contained within.
- Convection, evaporation, and radiation all squander heat from the solar pond’s UCZ.
- There is no insulating material between the SGSP and the ground.

**Fig 5:**
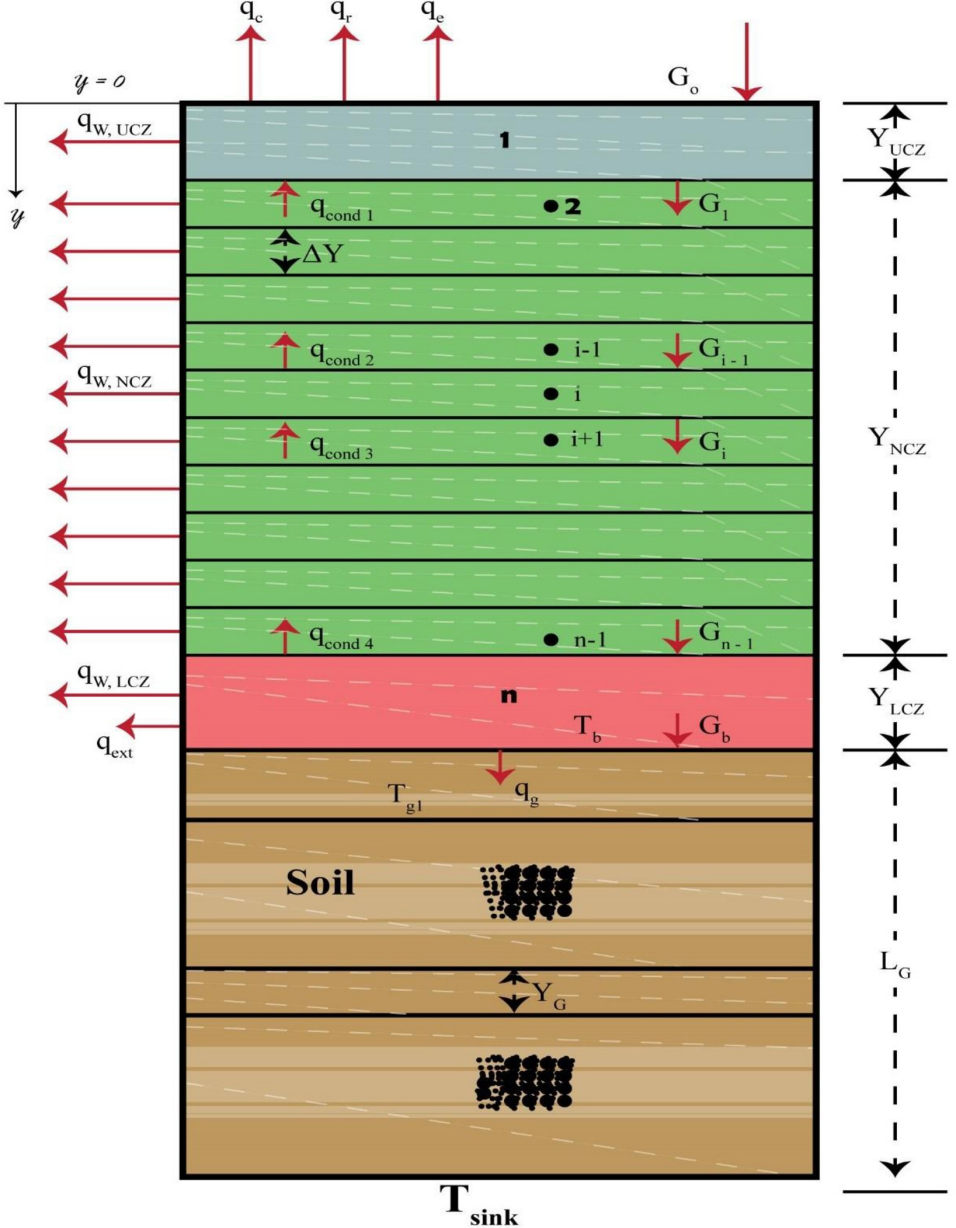
Mathematical models of solar pond and ground.

*2*.*2*.*1*.*2 SGSP heat transfer model*. As shown in Fig 5, vertical measurements are taken in the coordinate system with (y) being positive in the downward direction and y = 0 at the pond’s surface. The solar pond model created is based on the energy balance of a liquid layer. The energy input and outflow in the layer are equivalent to the energy loss and buildup over time. The pond and the ground underneath it are split into strata that extend to the water table. The LCZ and UCZ layers are regarded as a single layer, while the NCZ and ground are split into several levels. The energy balance for each layer is generated for each time step of the simulation. The energy conservation is defined as follows, based on the energy balances for UCZ, NCZ, and LCZ.

Current development in the SGSP may be seen as a two-dimensional transient conduction system for internal heat production. Thermodynamics within the pond and the ground below it are described using an implicit finite difference approach. As a result, the domain is split into many horizontal levels, each with its own set of attributes. The model assumes that LCZ and UCZ are Convection zones, which means they have a consistent temperature distribution and are considered a single layer. NCZ and the earth, on the other hand, are separated into many thin layers. Due to the lack of salt diffusion inside the pond, the overall thickness of the layers of the solar pond stays constant throughout time. The following are the characteristics of saltwater [4]:

$$k_{i}=0.5553-0.0000813\left(\frac{s_{i}}{100}\rho_{w}\right)+0.0008({T_{i}}^{n}-20)$$ (2.1)

$$\rho_{i}=998-0.65\left(\frac{s_{i}}{100}\rho_{w}\right)+0.0008({T_{i}}^{n}-20)$$ (2.2)

$$C_{P,i}=4180-4.396\left(\frac{s_{i}}{100}\rho_{w}\right)+0.0048{\left(\frac{s_{i}}{100}\rho_{w}\right)}^{2}$$ (2.3)

Where,

*ρ*_*w*_ = 1000 *kgm*^−3^ (Density of freshwater)

The thermal evolution within NCZ is described as [8]

$${\rho_{i}}^{n}{C_{P,i}}^{n}{\Delta z}_{i}\left(\frac{{T_{i}}^{n+1}-{T_{i}}^{n}}{\Delta t}\right)={q_{c,i}}^{n+1}+{q_{c,i-1}}^{n+1}+G_{i}-q_{w,i}-G_{i+1}$$ (2.4)

It is assumed that the solar radiations flowing through the water stream are exponential and handled in two distinct ways, whether they come from natural or artificial sources, and as a result [8]:

$$G(z)=\left\{ \begin{array}{c} (1-\alpha )(1-\beta )G_{o}\exp\left(-\eta z\right),\;for\;artificial\;lights\\ (1-\alpha )G_{o}\sum_{j=1}^{4}\left(1-\beta_{j}\right)\exp\left(-\eta_{j}z\right),\;for\;natural\;lights \end{array}\right.$$ (2.5)

Where,

*β and* η are the absorption and extinction coefficients for the multiple wavelength ranges of natural light.

*α* (*albedo*) is the fraction of incident radiations, reflected towards the atmosphere, and based on prior research works; it is anticipated to be 0.08 [19, 48].

The reduction in shortwave radiations is described by η as going deep inside the water column. *β* corresponds to the fraction of longwave radiation absorbed as it passes through the first few millimeters of water’s surface. However, when radiations originate from natural sources, the Rabl and Nielsen equation may explain the decrease in intensity and are evaluated for the different wavelength ranges in the spectrum [49].

The following version of Fourier’s law is used to calculate heat conduction from the i^th^ layer to the ground.


$${q_{C,i}}^{n+1}=\left(\frac{k_{i+1}+k_{i}}{2}\right)\left(\frac{{T_{i}}^{n+1}-{T_{i+1}}^{n+1}}{{\Delta z}_{i}}\right)$$ (2.6)

At the pond’s surface, the heat flow is determined by evaporation loss (q_e_), sensible heat (q_s_), and net longwave radiations (q_l_). Thus, the higher convective zone’s energy balance may be computed using the following equation [8]:

$${\rho_{U}}^{n}{C_{P,U}}^{n}z_{U}\left(\frac{{T_{U}}^{n+1}-{T_{U}}^{n}}{\Delta t}\right)={q_{C,U}}^{n+1}+G_{U}-G_{U+1}-q_{e}-q_{l}-q_{s}-q_{w,U}$$ (2.7)

Additionally, for the lower convective zone, the energy balance is as follows:

$$\rho_{L}C_{P,L}z_{L}\left(\frac{{T_{L}}^{n+1}-{T_{L}}^{n}}{\Delta t}\right)=q_{C,L-1}+I_{L}-q_{w,L}-q_{g}-q_{ext}$$ (2.8)

Where,

q_ext_ denotes the heat extracted from the lower zone.

q_g_ is the amount of heat lost to the ground through convection and is calculated as [50]:

$$q_{g}=h_{1}({T_{L+1}}^{n+1}-{T_{L}}^{n+1})$$ (2.9)

Here,

h_1_ is the convection heat coefficient between the LCZ and the ground, which is assumed to be constant at 78.12 Wm^-2^K^-1^.

So, the energy balance for k^th^ layer of the ground is:

$$\rho_{k}C_{P,k}{\Delta z}_{k}\left(\frac{{T_{k}}^{n+1}-{T_{k}}^{n}}{\Delta t}\right)=q_{c,k}+q_{c,k-1}+q_{w,k}$$ (2.10)

*2*.*2*.*1*.*3 Boundary conditions*. Relative humidity, wind speed, solar radiation, and air temperature are computed for the area around the solar pond, which may be used to determine heat fluxes at the upper boundary condition. The energy flux for the N^th^ layer is used to demonstrate the lower boundary conditions for the domain region *q*_*N*_ [8]

$${q_{N}}^{n+1}=\left\{ \begin{array}{c} K_{N}\frac{\left({T_{g}}^{n}-{T_{N}}^{n}\right)}{{\Delta z}_{N}},lower\;boundary\;limits\;with\;ground\\ h_{2}({T_{w}}^{n}-{T_{N}}^{n}),lower\;boundary\;limit\;with\;ground\;water\;table \end{array}\right.$$ (2.11)

Where,

h_2_ = 185.85 Wm^-2^K. [47] (Convective heat coefficient acting as a sink)

This energy may represent the interface between the groundwater table and the earth at a particular depth in the ground. If the domain imposes constraints on the ground, heat flow will be convective.

The energy flux q_w_ across the solar pond’s perimeters is utilized as the lateral boundary condition [51].


$${q_{W,i}}^{n+1}=\left\{ \begin{array}{c} U_{W,i}({T_{a}}^{n}-{T_{i}}^{n}),for\;unburied\\ U_{W,i}({T_{avg}}^{n}-{T_{i}}^{n}),for\;buried \end{array}\right.$$ (2.12)

For unburied SGSP, equation 4.12 (a) shows loss of heat from the pond to the surrounding atmosphere, whereas 4.12 (b) shows convective flux between the perimeter and a given distance x_g_ for buried SGSP, where heat flow from the pond is ignored, and the ground temperature is assumed to be constant as the average annual temperature T_avg_ [51].

The convective heat coefficient UW is assumed to be the reciprocal sum of the heat transfer resistance and the boundary for known temperature.


$$U_{W,i}=\frac{1}{\sum_{m=1}^{M}R_{m,i}}=\frac{1}{\sum_{m=1}^{M}\frac{x_{m}}{k_{m}}}$$ (2.13)

Where, M is the total number of materials, m denotes the various materials, and R_m_ denotes the conduction heat transfer resistance.

When the ground is considered a single layer, the heat flow is calculated as [51]:

$$q_{g}=U_{g}({T_{L}}^{n+1}-T_{gw})$$ (2.14)

Where, U_g_ is the ground’s total heat transfer coefficient between the pond and the groundwater table, computed using equation (4.13).

*2*.*2*.*1*.*4 Heat loss to the atmosphere*. The loss of heat due to evaporation is given by [52, 53]:

$$q_{e}={\left({q_{free}}^{2}+{q_{forced}}^{2}\right)}^{\frac{1}{2}}$$ (2.15)

Where *q*_*free*_ is free convection and *q*_*forced*_ is forced convection and are given by:

$$q_{free}=\left\{ \begin{array}{l} 2.7*10^{-2}{\left(T_{wv}-T_{av}\right)}^{\frac{1}{3}}*(e_{sw}-e_{a}),if\;T_{wv}>T_{av}\\ 0\;,if\;T_{wv}\leq T_{av} \end{array}\right.$$ (2.16)

$$q_{forced}=3.1*10^{-2}U_{2}(e_{sw}-e_{a})$$ (2.17)

$$T_{wv}=\frac{T_{w}}{1-\frac{0.378}{P_{atm}}e_{sw}}$$ (2.18)

$$T_{av}=\frac{T_{a}}{1-\frac{0.378}{P_{atm}}e_{a}}$$ (2.19)

$$e_{sw}=2.718*10^{10}\exp\left(\frac{-4157}{T_{w}+239.34}\right)$$ (2.20)

$$e_{a}=hr*e_{sat}$$ (2.21)

$$e_{sat}=2.718*10^{10}\exp\left(\frac{-4157}{T_{a}+239.34}\right)$$ (2.22)

$$P_{atm}=101300*\exp\left(\frac{-h}{8.200}\right)$$ (2.23)

*2*.*2*.*1*.*5 Longwave radiation flux (Q*_*l*_*)*. It deals with the difference between the longwave radiations emitting from water (*q*_*lw*_) and atmosphere (*q*_*la*_), which is given by:

$$q_{l}=q_{lw}-q_{la}$$ (2.24)

And,

$$q_{lw}=\varepsilon_{w}\sigma {\left(T_{u}+273.15\right)}^{4}$$ (2.25)

$$q_{la}=\varepsilon_{a}\sigma {\left(T_{a}+273.15\right)}^{4}$$ (2.26)

Here,

*ε*_*a*_ is the emissivity of air and is given by [54]:

$$\varepsilon_{a}=0.87-2.693*\exp\left(-2.693*10^{-5}e_{a}\right)$$ (2.27)

*2*.*2*.*1*.*6 Sensible heat flux*. Sensible heat flux is given as [55]:

$$q_{s}=1.5701*U_{2}(T_{w}-T_{a})$$ (2.28)

## 3. Results and discussion

The impact of various soil characteristics (sandy soil texture, soil type, and moisture content), the thickness of pond layers, and the water table level are examined.

### 3.1 Effect of soil type on temperature of LCZ

**Fig 6:**
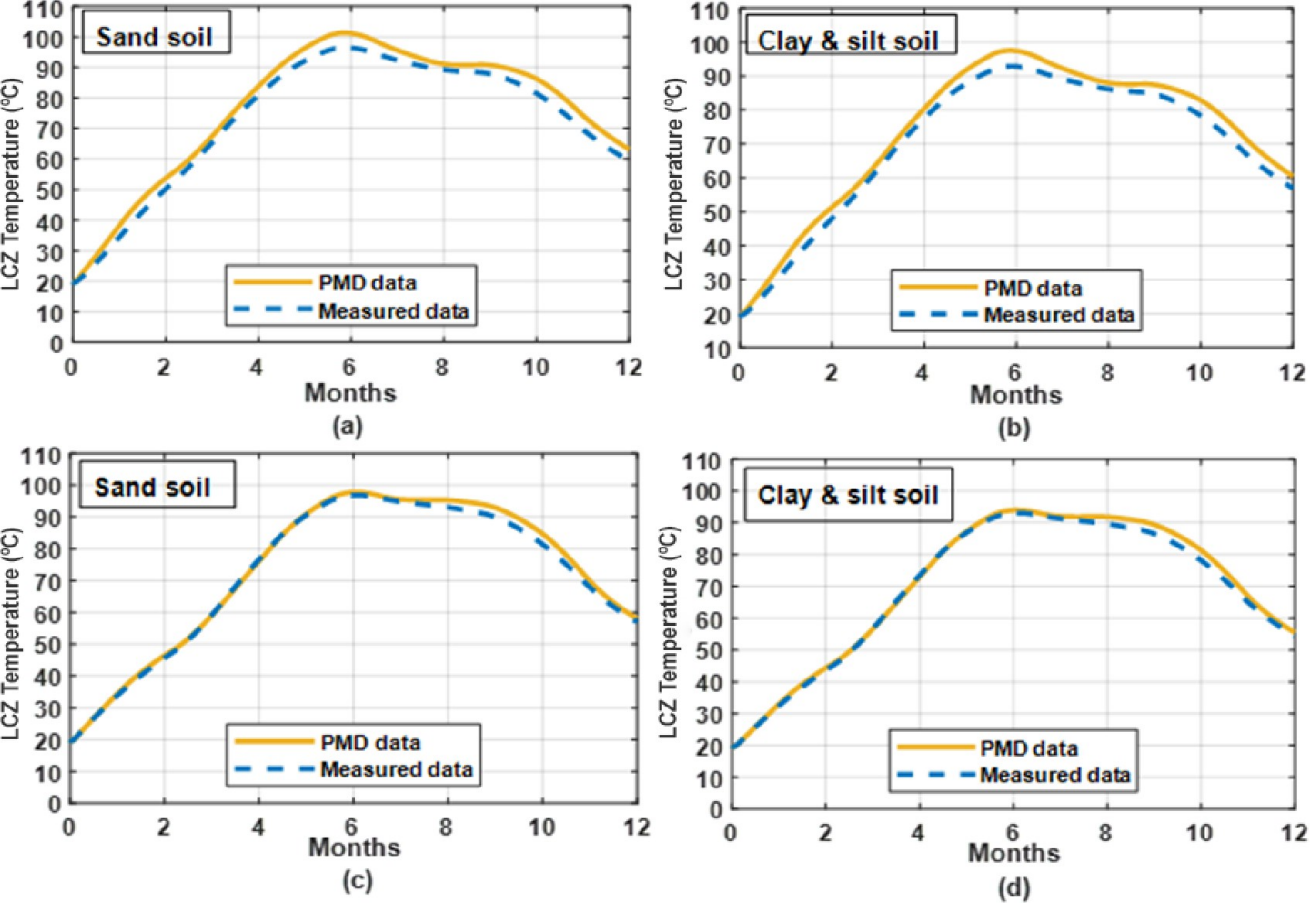
Annual LCZ temperature with a different type of soil under the solar pond for whole year simulation under PMD and measured climatic conditions of Lahore (a), (b) and Islamabad (c), (d).

Fig 6 depicts the LCZ temperature using two different kinds of soil under the solar pond: sandy soil from [56] and silt soil and clay from [57]. In this figure, we studied the effect of two different types of soil as sand soil and clay & silt soil under the climatic conditions of two different cities of Pakistan as Lahore and Islamabad. Under the solar pond, it is found that the temperature of LCZ is higher with sand soil than with clay and silt soil. It is consistent with the fact that clay and silt soils have a greater heat conductivity than sandy soils. As a result, heat loss from the SGSP to the ground will rise when the soil under the pond is mostly silt soil and clay and will decrease when the soil beneath the pond is predominantly sand soil. The maximum temperature achieved in LCZ is 101.30°C under the climatic conditions of Lahore by using sand soil and 97.54°C for clay & silt soil as given in Table 2. Therefore, it was recommended to construct the pond on soil with low thermal conductivity.

**Table 2:** Results summary for maximum LCZ temperature under different climatic and soil conditions.

Parameters	Variation of Parameters	PMD data Lahore	Measured data Lahore	PMD data Islamabad	Measured data Islamabad
Soil Type	Sand soil	101.30	96.47	97.77	96.83
	Clay and silt soil	97.54	92.88	93.94	93.01
Moisture Content	Clay and silt soil (5%)	94.94	90.40	91.42	90.59
	Clay and silt soil (10%)	90.58	86.23	87.04	86.18
Soil Texture	Fine sand dry	108.35	103.21	104.86	103.80
	Coarse sand dry	106.73	101.66	103.15	102.21
Depth of water table (Lg)	Lg = 2 m	89.69	85.24	86.34	85.10
	Lg = 4 m	96.48	91.86	92.94	91.89
	Lg = 6m	97.35	92.70	93.77	92.78
	Lg = 8 m	97.54	92.87	93.96	92.99
Thickness of UCZ	UCZ = 0.1 m	99.47	94.30	95.45	94.59
	UCZ = 0.3 m	97.55	92.48	93.57	92.72
	UCZ = 0.5 m	95.69	90.70	91.75	90.87
	UCZ = 0.7 m	93.87	88.98	89.95	89.07
Thickness of NCZ	NCZ = 0.5 m	95.65	90.30	91.52	90.45
	NCZ = 1.0 m	97.54	92.55	93.73	92.66
	NCZ = 1.5 m	99.65	94.25	95.50	94.23
	NCZ = 2.0m	101.10	95.93	96.96	95.36
Thickness of LCZ	LCZ = 0.5 m	99.14	94.15	95.60	94.37
	LCZ = 1.5 m	97.55	92.63	94.15	92.84
	LCZ = 2.5 m	95.65	90.71	92.24	90.88
	LCZ = 3.5 m	93.63	88.87	90.28	88.92
Heat Extraction	No Load	97.55	92.86	93.95	93.01
	10 W/m2	93.80	89.15	90.24	89.30
	20 W/m2	90.11	85.45	86.51	85.57
	30 W/m2	86.37	81.73	82.79	81.84

### 3.2 Effect of moisture content on temperature of LCZ

**Fig 7:**
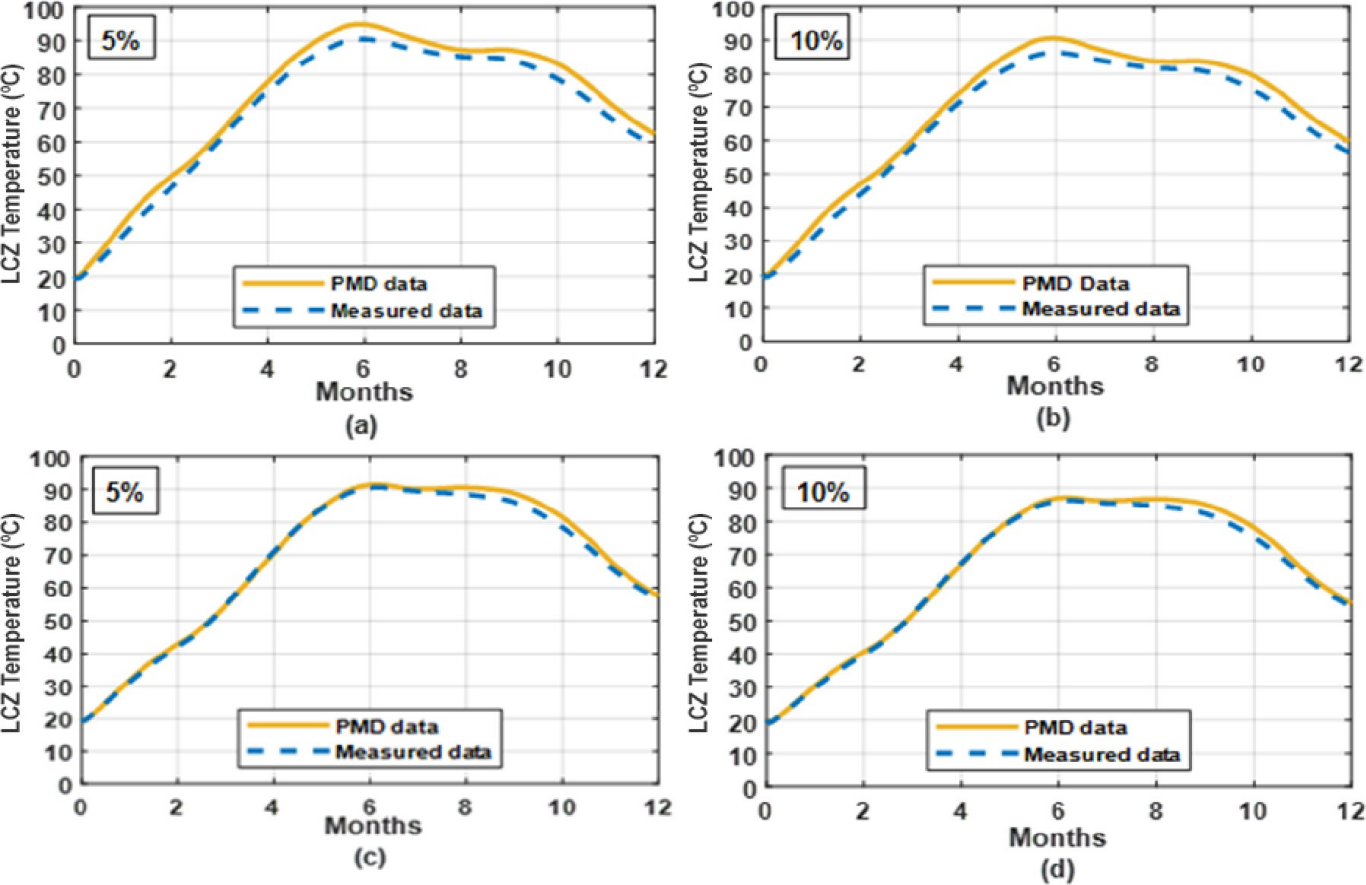
LCZ temperature with different moisture contents in soil under the solar pond for whole year simulation under PMD and measured climatic conditions of Lahore (a), (b) and Islamabad (c), (d).

Fig 7 depicts the effect of soil moisture on the solar pond’s lower convective region temperature for chosen soil (clay and silt soil). As can be observed, increasing the soil moisture content by 5–10% decreases the SGSP’s LCZ temperature. Increasing the moisture level increases the interaction between solid particles by generating thermal bridges and creating a continuous route for heat transmission. It is observed that the hot water temperature with 5% moisture content for clay and silt soil under the solar pond is higher than the hot water inlet temperature with 10% moisture content for clay and silt soil. The reason behind that is the soil underneath the solar pond with 10% moisture content has high thermal conductivity and that leads to increase the heat loss to the ground and decrease the LCZ temperature achieved by the pond. The maximum temperature difference in LCZ is 4.36°C under the climatic conditions of Lahore by using 5% -10% moisture content for clay & silt soil as given in Table 2. Therefore, it was recommended to construct the pond on soil with low moisture content.

### 3.3 Effect of sandy soil texture on temperature of LCZ

**Fig 8:**
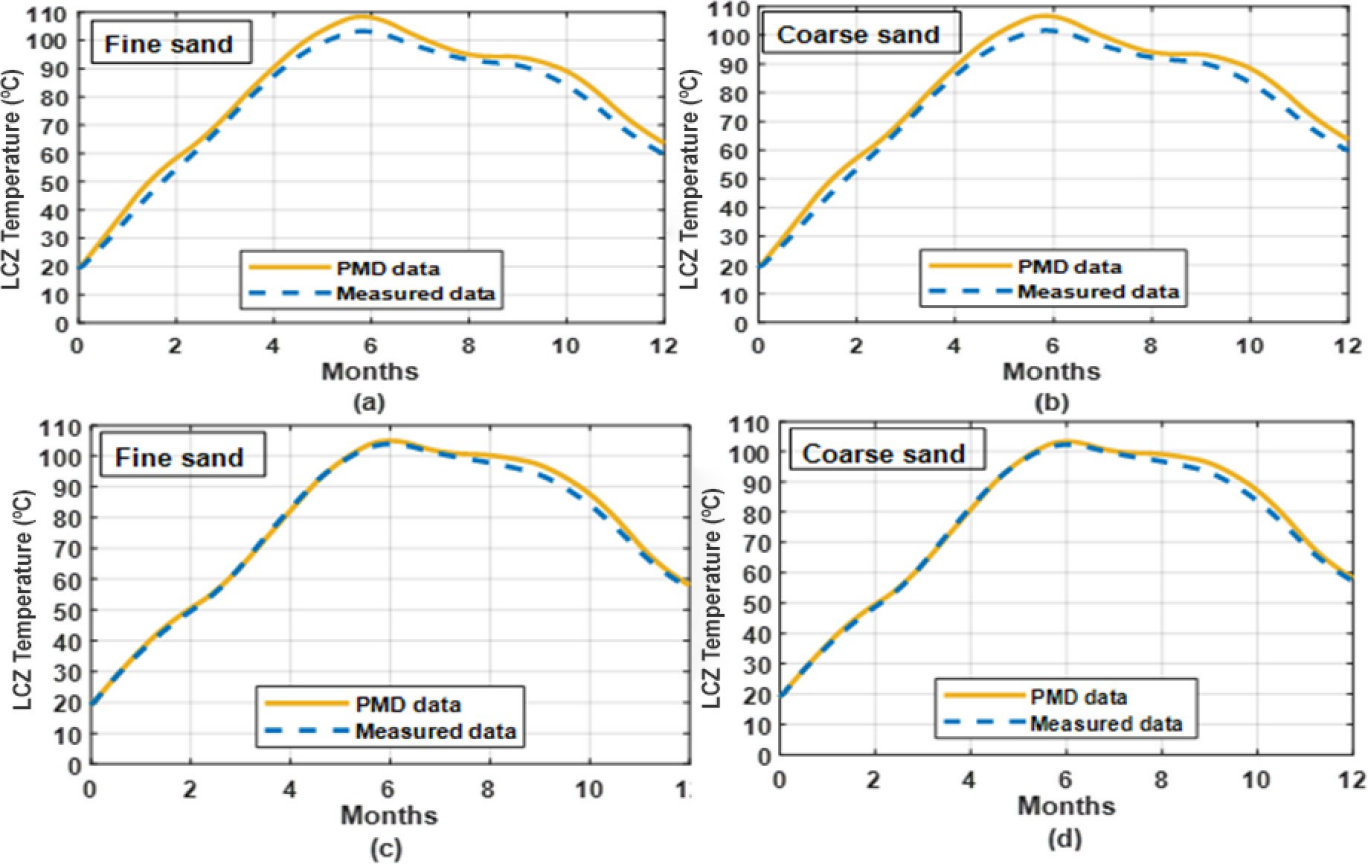
LCZ temperature with different sandy soil texture under the solar pond for whole year simulation under PMD and measured climatic conditions of Lahore (a), (b) and Islamabad (c), (d).

The effect of sandy soil texture on the LCZ temperature of SGSP at 6m water table level is shown in Fig 8. Coarse sand dry soils have a somewhat less significant impact on temperature as compared to fine dry soils. Under the climatic circumstances of Lahore and Islamabad, the temperature difference between the two soil textures is negligible. Due to the coarse dry sand soils and poor heat conductivity of the fine, these temperatures were reached at a very high level in the SGSP model used in this research. The maximum temperature achieved in LCZ is 108.35°C under the climatic conditions of Lahore for fine sand dry soil and 106.73°C for coarse sand dry as given in Table 2. There is a little difference in temperature as 1.62°C for LCZ due to less difference in thermal properties.

For coarse and fine sand, the values are comparable with minor variations, which is because the soil diffusivity for coarse and fine sand is almost the same. The site with fine sand soil is one of the finest locations for the solar pond. It is concluded that the site which has dry, fine sand soil is one of the best choices to construct the solar pond on it.

### 3.4 Effect of the water table depth on temperature of LCZ

At the non-insulated bottom of the solar pond, heat is lost from solar pond to the ground. The magnitude of heat loss depends on the soil thermal properties and on the depth of the water table underneath the solar pond, which act as a heat sink. The effect of varying the depth of the water table under solar pond for clay and silt soil has been investigated. The values are 2m, 4m, 6m, 8m measured from the bottom of the solar pond.

**Fig 9:**
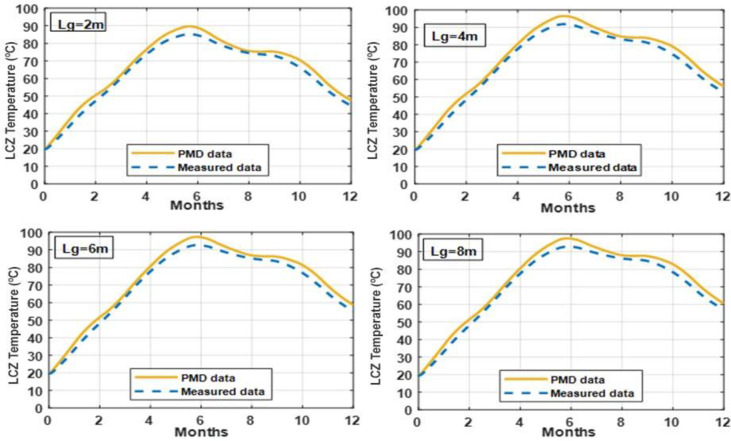
LCZ temperature with different depths of water table under the solar pond for whole year simulation under PMD and measured climatic conditions of Lahore.

**Fig 10:**
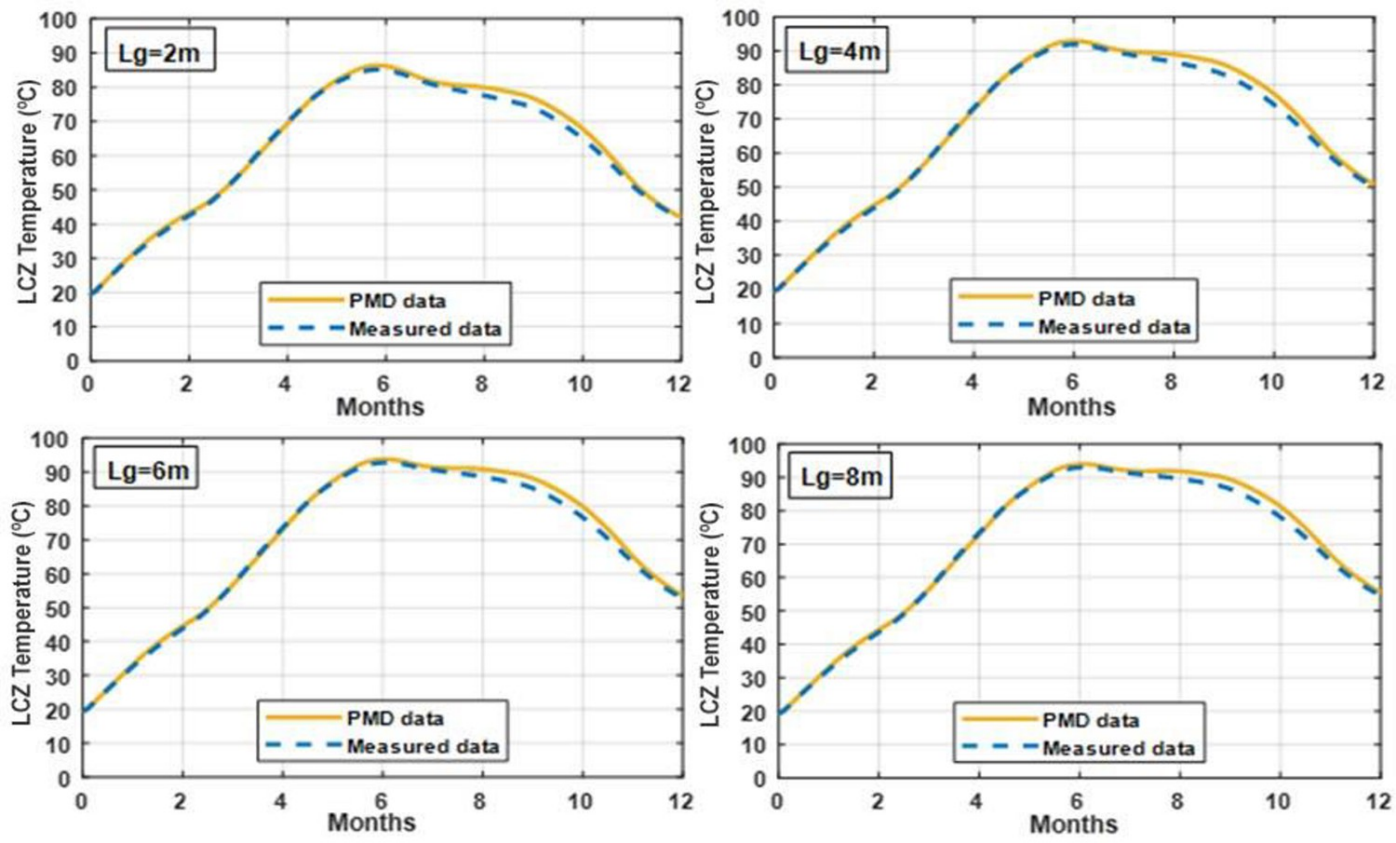
LCZ temperature with different depths of the water table under the solar pond for whole year simulation under PMD and measured climatic conditions of Islamabad.

Figs 9 and 10 illustrate the impact of water table depth on the LCZ temperature in a solar pond with a salinity gradient and clay and silt soil. It demonstrates that the water table depth has a substantial impact on the SGSP’s LCZ temperature. It is self-evident that the deeper the water table, the greater the LCZ temperature and, therefore, the better the pond performance. It is to minimize heat loss to the earth. The water table depth between 2 and 6 meters on the LCZ temperature is significant, while the water table level between 6 and 8 meters has a negligible effect on the LCZ temperature. As a result, the minimal water table depth beneath the solar pond in this research was selected to be 5–6 meters. It is recommended to search for a site with deep water table under soil before construct the pond.

### 3.5 Effect of varying UCZ thickness on temperature of LCZ

**Fig 11:**
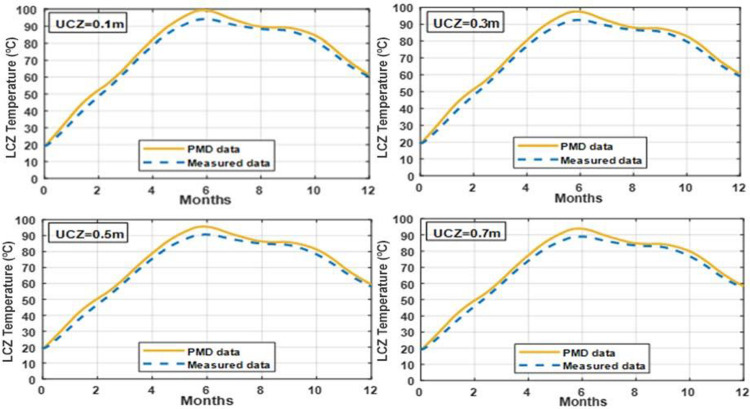
Effect of different thickness of UCZ on the temperature of LCZ with NCZ = 1 m and LCZ = 0.7 m under PMD and measured climatic conditions of Lahore.

**Fig 12:**
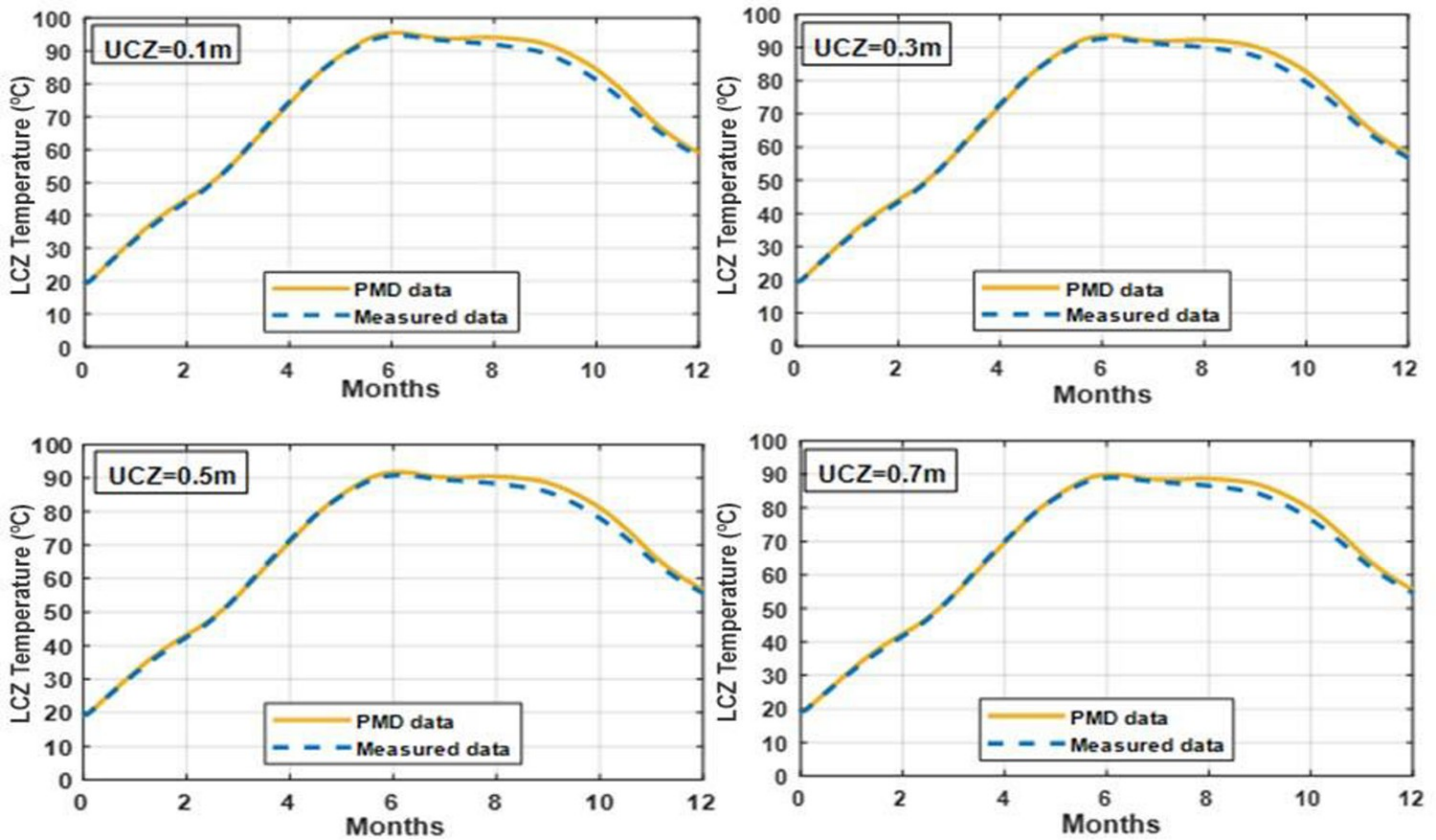
Effect of different thickness UCZ on the temperature of LCZ with NCZ = 1 m and LCZ = 0.7 m under PMD and measured climatic conditions of Islamabad.

Figs 11 and 12 illustrate the impact of changing UCZ thickness on the temperature of the LCZ with NCZ = 1.0 m and LCZ = 0.7 m in clay and silt soil. Increasing the UCZ thickness is expected to reduce the amount of solar radiation that penetrates in to the NCZ and LCZ, thereby reducing the LCZ temperature and useful heat collected. As seen by this graph, increasing UCZ thickness reduces UCZ temperature in the lower convective zone. The dense UCZ zone decreases the amount of solar radiation that reaches the bottom of the SGSP, lowering the temperature of LCZ and solar pond performance. A 0.1 m thickness, on the other hand, is insufficient to protect the pond from the effects of rain, wind mixing, and evaporation. The UCZ temperature is not expected to be significant affected, as the UCZ is well mixed and in contact with the ambient air. As a result, a practical minimum thickness of (0.3 m) is suggested.

### 3.6 Effect of varying NCZ thickness on temperature of LCZ

The NCZ in the pond plays an important role in determining the actual thermal performance. It works as thermally insulating layer between UCZ and LCZ. Increasing the NCZ thickness will have two opposing effects: (1) Improving the thermal insulation, hence tending to increase the LCZ temperature and (2) reducing the amount of solar radiation which reaches the LCZ, hence tending to reduce the LCZ temperature.

**Fig 13:**
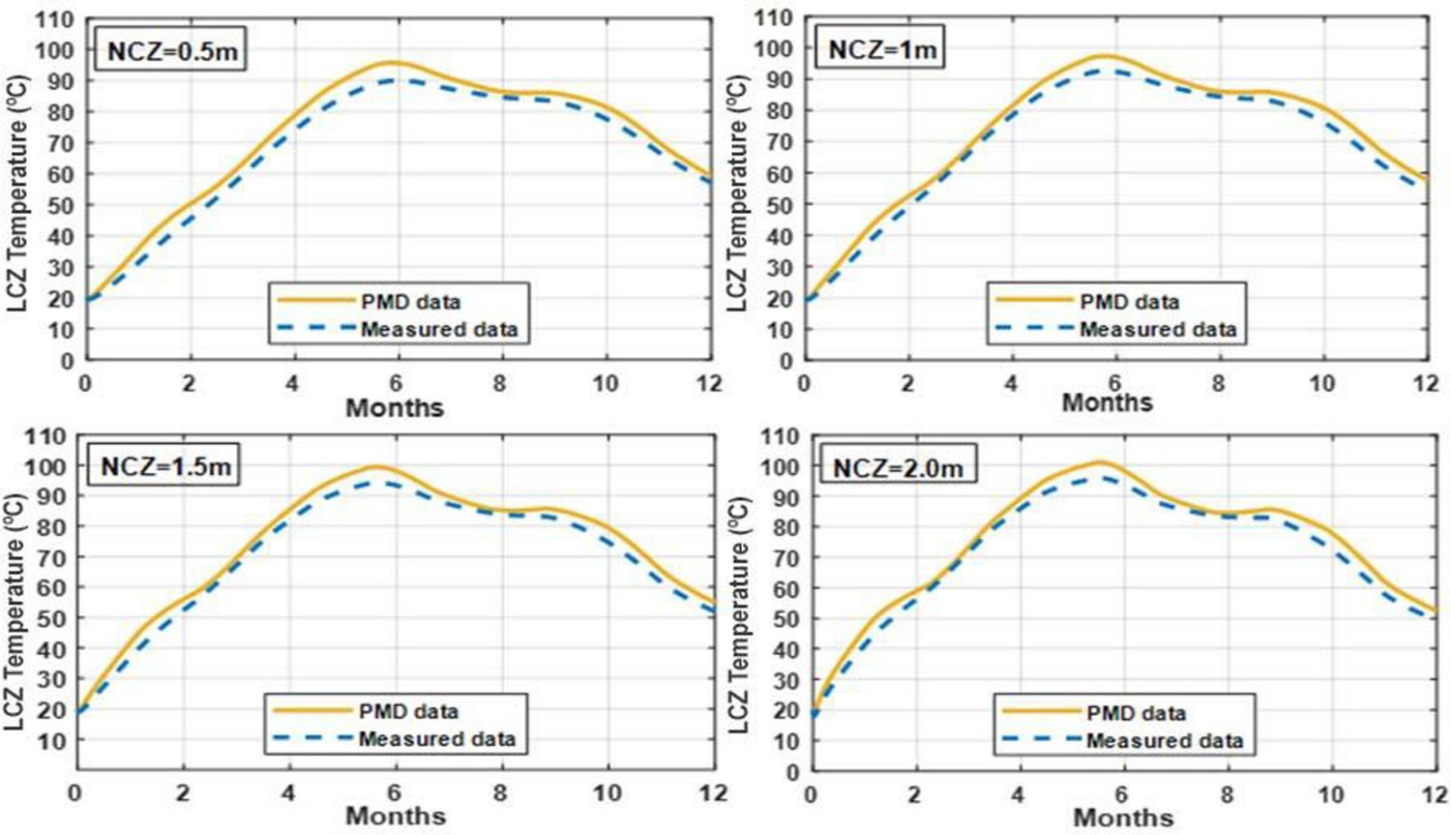
Effect of different thickness of NCZ on the temperature of LCZ with UCZ = 0.3 m and LCZ = 0.7 m for whole year simulation under PMD and measured climatic conditions of Lahore.

**Fig 14:**
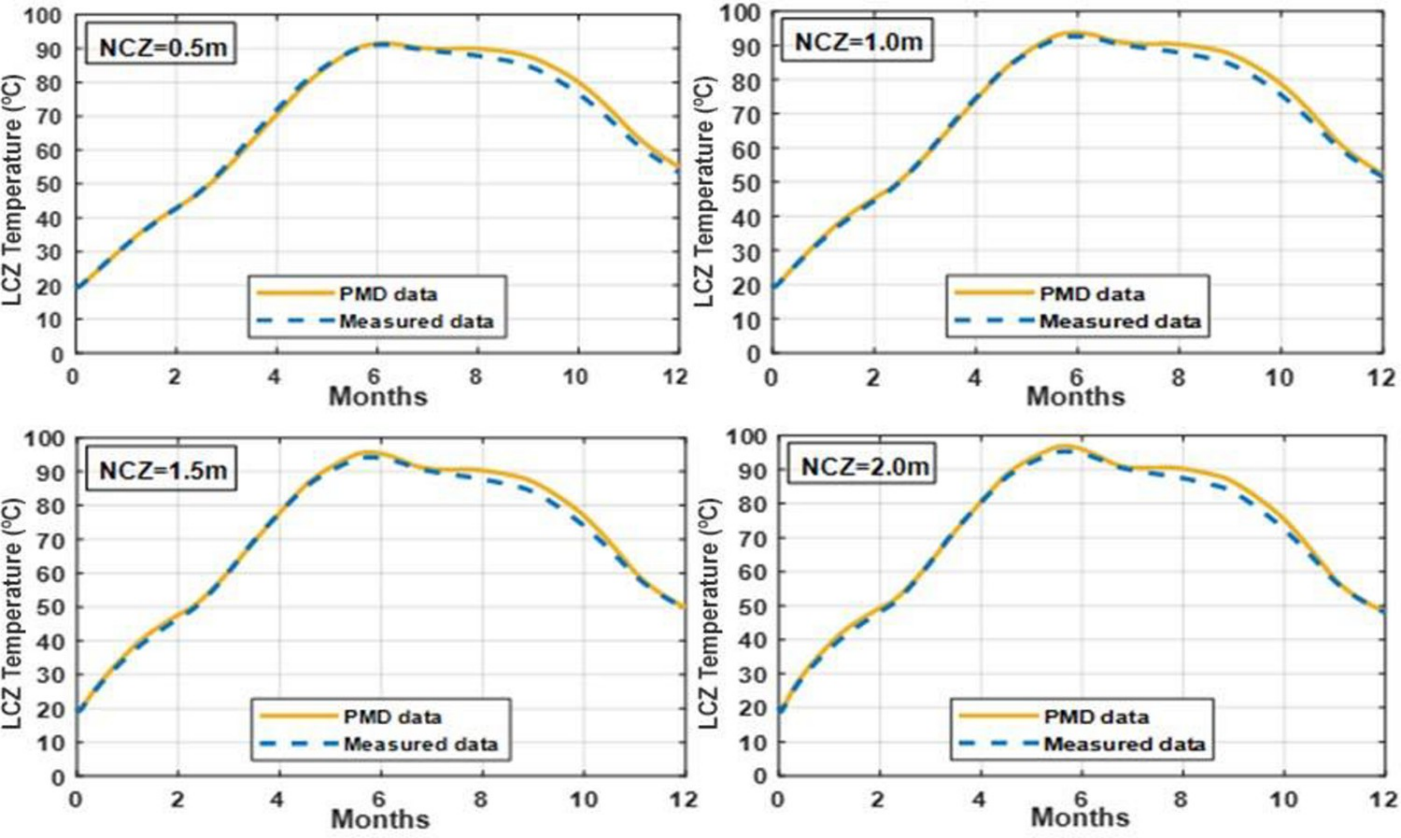
Effect of different thickness of NCZ on the temperature of LCZ with UCZ = 0.3 m and LCZ = 0.7 m for whole year simulation under PMD and measured climatic conditions of Islamabad.

Figs 13 and 14 demonstrate the impact of changing the thickness of the NCZ on the temperature of the bottom zone in clay and silt soils. The greatest LCZ temperature is seen for the thickest NCZ, indicating that even at the highest thicknesses tested, the benefit of decreased heat loss from the LCZ layer outweighs the disadvantage of lower solar gain to the LCZ layer.

### 3.7 Effect of varying LCZ thickness on temperature of LCZ

The useful heat is stored and extracted from the lower convective zone of the solar pond. This heat is the result of the energy balance for LCZ. Increasing the thickness of the LCZ will increase its thermal capacity, so it will take longer to heat up and longer to cool down.

**Fig 15:**
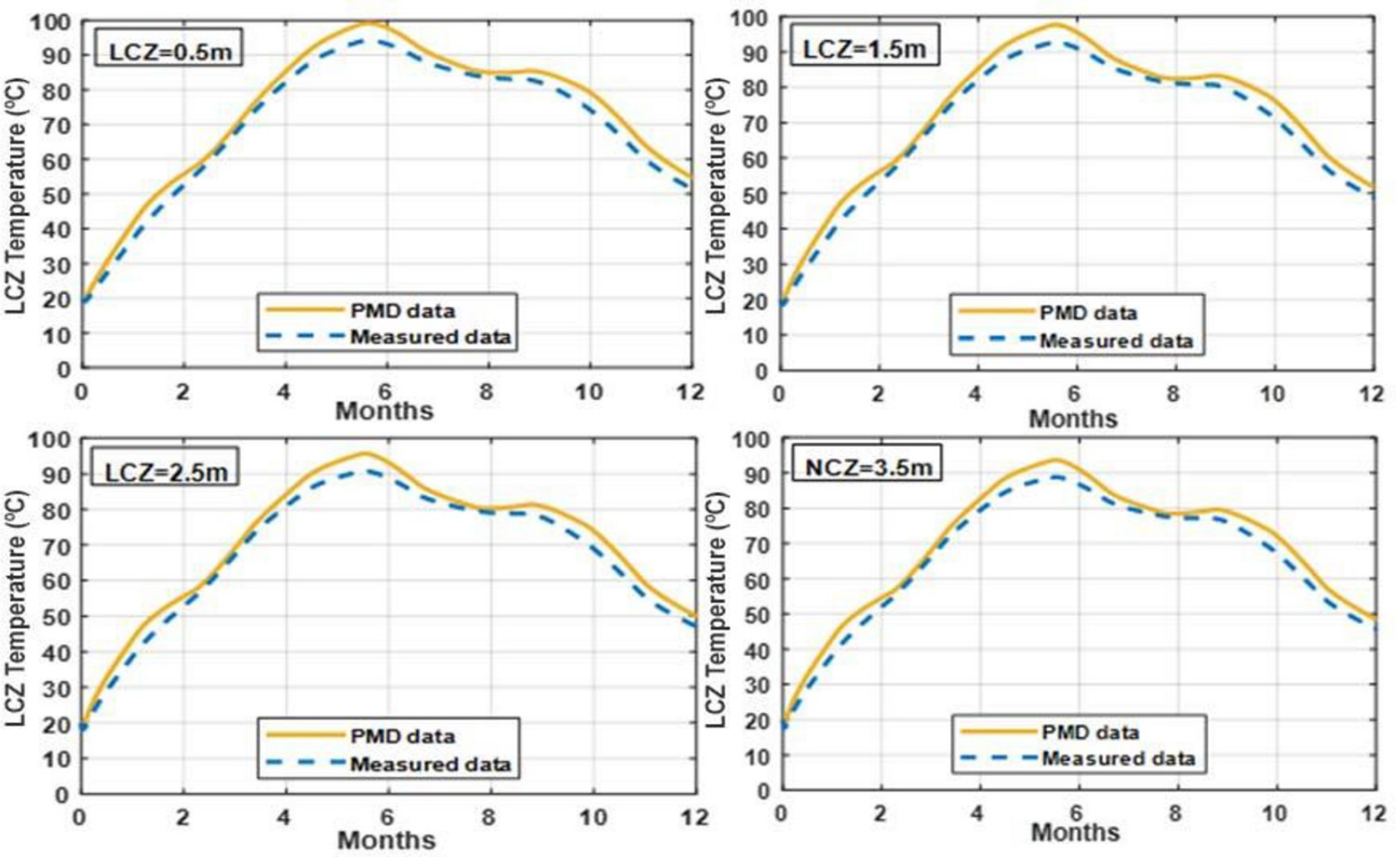
Effect of different thickness of LCZ on the temperature of LCZ with UCZ = 0.3m and NCZ = 1.0m under PMD and measured climatic conditions of Lahore.

**Fig 16:**
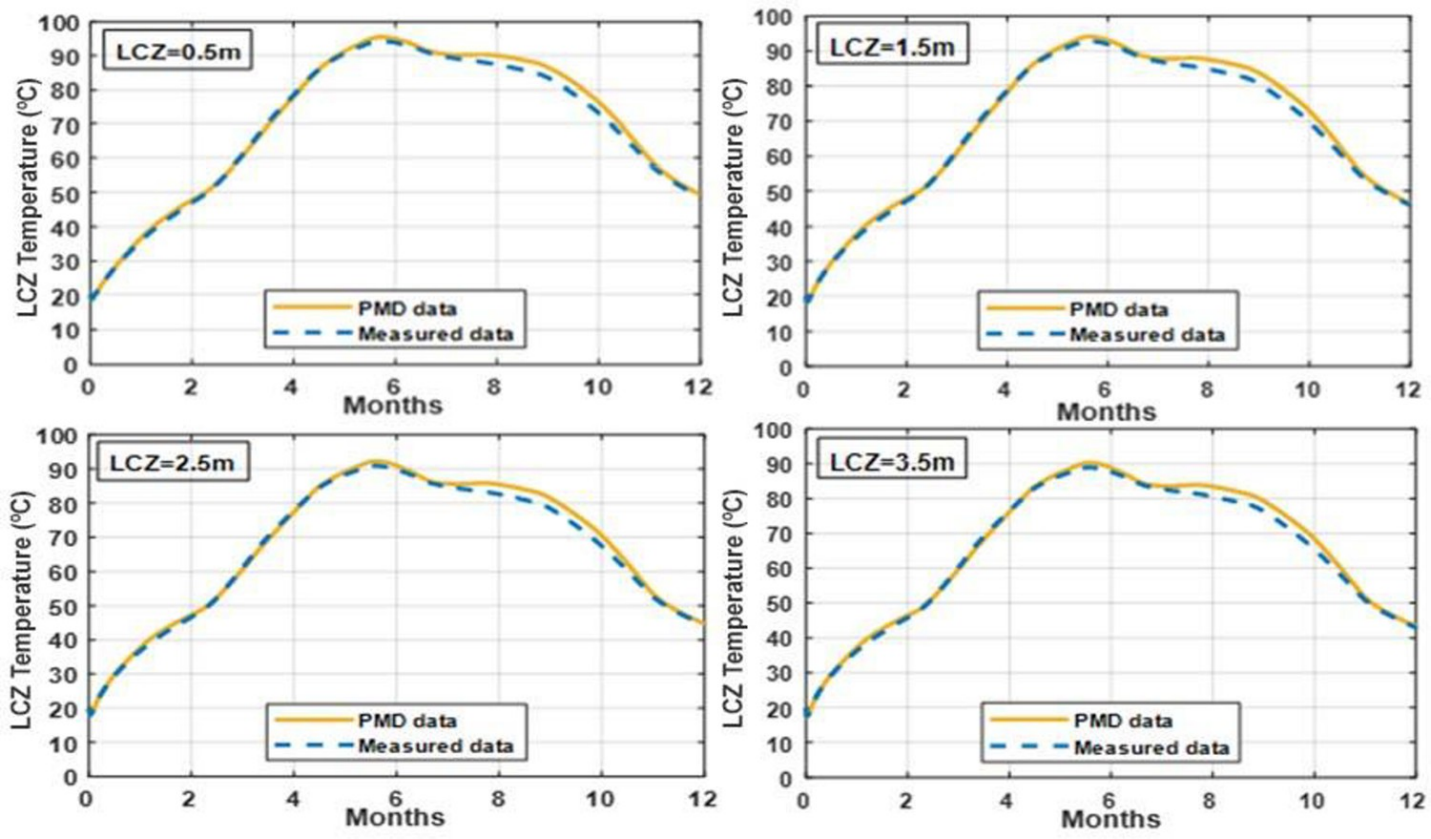
Effect of different thickness of LCZ on the temperature of LCZ with UCZ = 0.3m and NCZ = 1.0m under PMD and measured climatic conditions of Islamabad.

The temperature of the lower convective zone in clay and silt soils may be altered by changing the LCZ thickness, as shown in Figs 15 and 16. When a thicker layer of LCZ is used, the temperature of the LCZ is somewhat reduced. Different thicknesses of 0.5m, 1.5m, 2.5m, and 3.5m increase the temperature differential between the LCZ to a lesser degree. A thickness of 0.5 m is preferable to 1.5 m by a slight margin. Still, there will be some heat trapped inside the LCZ; therefore, 0.7 m is the optimal LCZ thickness. The 0.7 thickness of the LCZ may be suggested to achieve more heat storage to compensate for decreased solar radiation during the summer heatwave or unexpected weather.

### 3.8 Energy rate extraction

**Fig 17:**
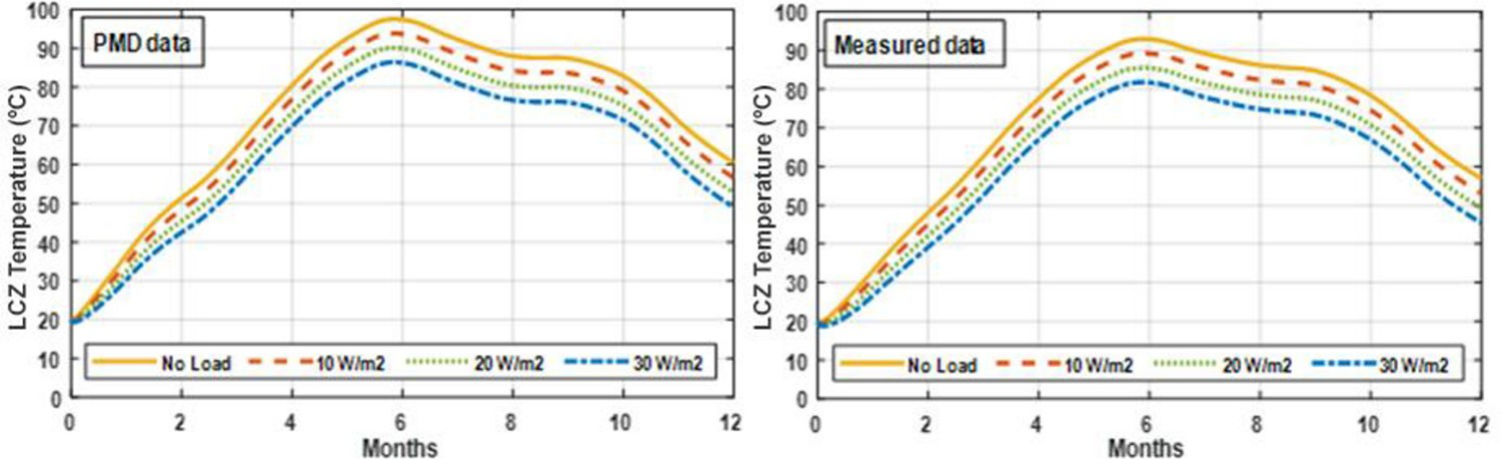
Energy rate extraction from the pond under PMD and measured climatic conditions of Lahore.

**Fig 18:**
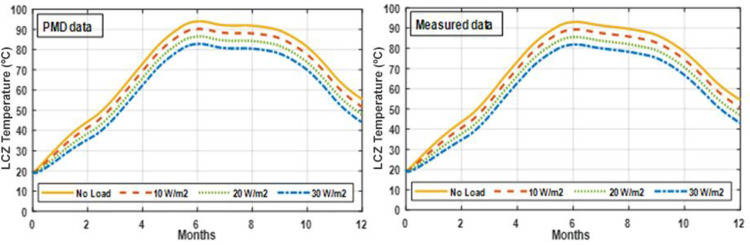
Heat extraction from the pond under PMD and measured climatic conditions of Islamabad.

Simulations are run over a year-long period in this study. Extracted energy from the SGSP using MATLAB code with various amounts of heat extraction from the SGSP. Figs 17 and 18 depict the yearly temperature profile of the LCZ for each month with varying loads. The highest temperature was recorded in June and then progressively dropped to a level equal to or greater than the pond’s original temperature measurement. The LCZ temperature is determined by the starting temperature, the quantity of solar radiation absorbed by the LCZ, the monthly ambient temperature, and the amount of energy produced from the SGSP. The temperature of the LCZ lowers when the load is increased.

### 3.9 Validation of the proposed model

This section compares the numerical results from the proposed study to the already published experimental studies for the purpose of validation. The proposed model used SGSPs with a surface area of 1m^2^, a depth of 0.3m for UCZ, 1m for NCZ, and 0.7m for LCZ. The numerical solution is written and solved in MATLAB for determining the time variations of the temperatures, as well as the thermal behavior of the pond. Results were simulated using the proposed numerical model of SGSP under the climatic and soil conditions of Lahore, Pakistan. The simulation results were verified against the temperature distributions of an experimental solar pond built at the Kuwait Solar Energy Department’s KISR facility that have an 8 m^2^ surface area with 0.2 m, 0.4 m, and 0.3 m for UCZ, NCZ, and LCZ, respectively [40]. The simulation results were also validated against the temperature distributions of an experimental solar pond built at the Urmia University, Iran that have an 4 m^2^ surface area with 0.2 m, 0.5 m, and 0.4 m for UCZ, NCZ, and LCZ, respectively [58].

**Fig 19:**
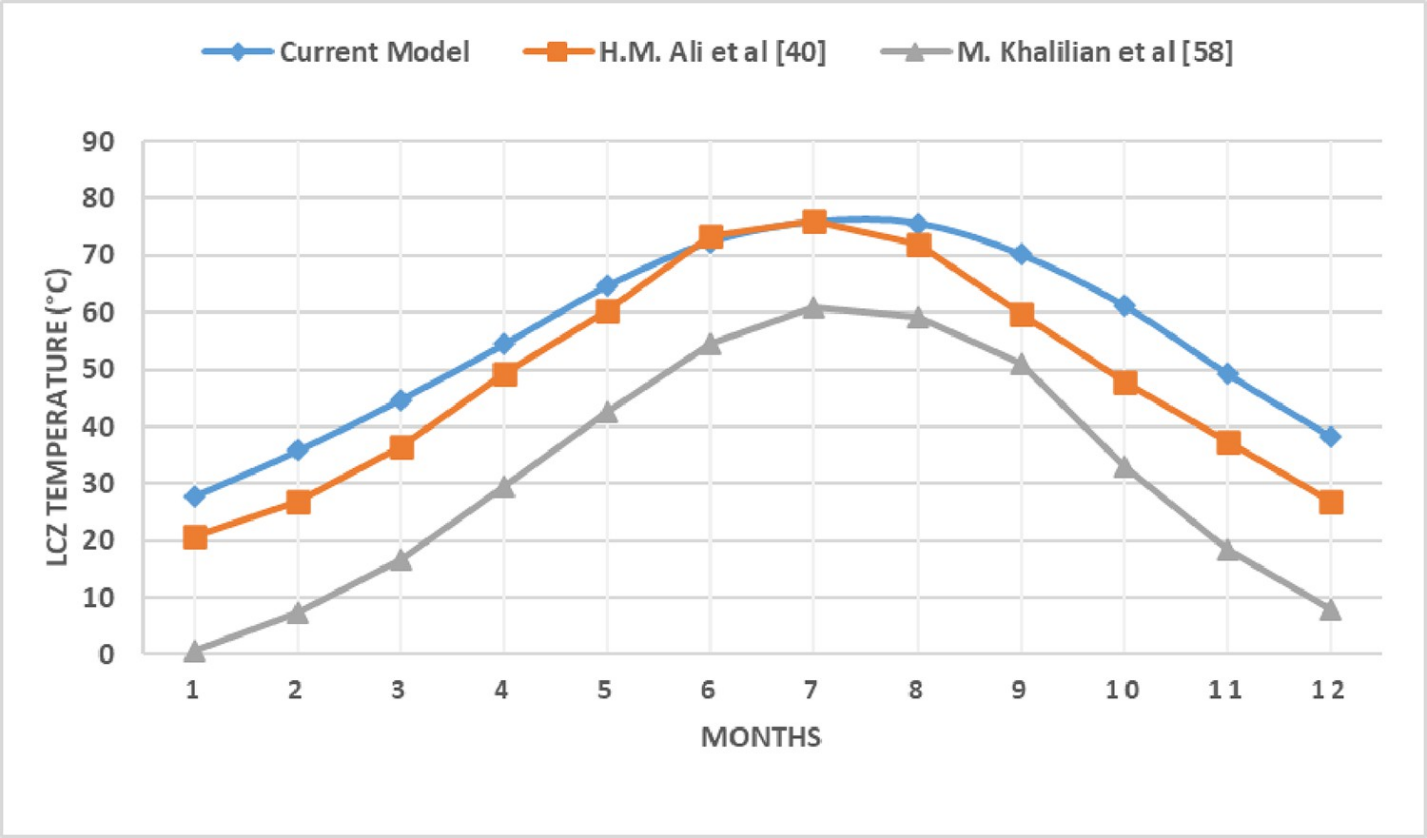
Validation of the proposed numerical model with experimental studies in [40, 58].

The temperature profiles of LCZ over a whole year extracted from the proposed numerical model and the experimental investigations of actual SGSPs are shown in Fig 19. The deviation in the temperature profiles of numerical and experimental studies is due to the difference in dimensions (surface area and thickness of UCZ, NCZ and LCZ), and the difference in soil and climatic conditions of each SGSP under comparison. However, It can be observed that overall temperature profile from the simulation results of the proposed model are in good agreement with the experimental results and are following the same trends.

### 3.10 Results summary

Table 2.

## 4. Economic analysis

Numerous businesses, such as pharmaceutical and food processing plants rely on high-temperature water for heating, cooling, and desalination processes. The temperature at which water must be boiled, ranges between 60°C and 90°C in a variety of endeavors. Solar ponds are an extremely efficient and cost-effective way to meet these industrial needs.

The maximum water temperature in the Lower Convective Zone (LCZ) of proposed solar pond was simulated to be 106.8°C. Heat energy was extracted from this hot water until water temperature drops down to 62.4°C, that is approximately the minimal water temperature to be used in most of the process industries. For the economic feasibility analysis of the proposed solar pond, price of extracted energy was calculated in terms of savings of carbon dioxide emissions and compared with the current unit price of electricity in Pakistan.

The economic analysis in Table 3 compares CO_2_ emissions and energy prices to the yearly output of an SGSP. The overall energy extraction rate from a 1m^2^ solar pond at a temperature of 62.48°C is 110 W/m^2^. The simulations generated a maximum yearly output of 963.6 kWh/m2/year from the solar pond, resulting in a reduction of 4336.2 kg CO_2_ emissions per year valued at $693.29 and a cost savings of 80$ per year if extracted energy is compared to the unit electricity price in Pakistan. Estimated cost of the proposed solar pond including the material cost, construction cost, labour cost and the maintenance cost are to be $1400. The payback time of a solar pond is calculated as the ratio of the pond’s installation expenses to the pond’s yearly savings. Payback period is estimated to be two years, based on savings of CO_2_ emissions and compared with the current unit price of electricity in Pakistan. According to this research, solar ponds are a technically and economically feasible solution for the industrial sector that requires hot water. Some of the important calculation parameters used for economic feasibility of proposed solar pond are given below:

1 KWh of energy produced by burning of natural gas = 4.5 Kg of CO_2_ emissions

Maximum energy extraction from proposed solar pond = 110 W/m^2^

8.76 kWh/m2.year = 1 W/m^2^

Annual Yield of energy from proposed solar pond = 110 W/m^2^*8.76 = 963.6 kWh/m^2^.year

Total annual CO_2_ emission = 4.5 * 963.6 = 4336.2 Kg

CO_2_ emission price per kg = 0.16$

Total annual CO_2_ emission price = 4336.2 * 0.16 = 693.29$/year

Electricity Unit (kWh) = 0.083$

Total annual electricity units price = 0.083*963.6 = 80$

**Table 3:** Analysis of saving costs of gas and electricity of domestic usage against annual yield of solar pond.

Energy rate Extraction (W/m2)	Temperature (°C)	Annual Yield (kWh/m2.year)	Gas		Electricity
			CO_2_ Emission (kg)	CO_2_ Emission Price ($)	Consumed Price ($)
0	108.36	0	0	0	0
10	103.98	87.6	394.2	$63.07	$7.27
20	99.95	175.2	788.4	$126.14	$14.54
30	95.74	262.8	1182.6	$189.22	$21.81
40	91.53	350.4	1576.8	$252.29	$29.08
50	87.31	438	1971	$315.36	$36.35
60	83.12	525.6	2365.2	$378.43	$43.62
70	78.9	613.2	2759.4	$441.50	$50.89
80	74.67	700.8	3153.6	$504.58	$58.16
90	70.48	788.4	3547.8	$567.65	$64.43
100	66.22	876	3942	$630.72	$72.71
110	62.48	963.6	4336.2	$693.29	$80

## 5. Conclusion

This research effort resulted in developing a mathematical model including heat and mass transport equations for three different SGSP zones. The mathematical model generates and analyses the temperature distribution and concentration profile distributions. The author concluded:

- Due to differences in thermal conductivity, sand soil has a higher LCZ temperature than clay and silt soil. In comparison to clay and silt soil, sandy soil has poor heat conductivity. Owing to the variation in heat conductivity between fine and coarse sand, the temperature of LCZ for fine sand dry is greater than for coarse sand dry.
- Clay and silt soil (5% moisture content) have a higher LCZ temperature than Clay and silt soil (10% moisture content). This discrepancy is caused by a change in heat conductivity caused by changes in moisture content. When the moisture content of the air rose, thermal conductivity increased.
- Due to its very poor heat conductivity, the overall solar pond performs best with fine sand.
- When the water table depth rises, the LCZ temperature is raised to minimize losses. The optimal depth of the water table is 5 to 6 m.
- When the thickness of NCZ drops, so does the temperature of LCZ. It increases when the thickness of the LCZ decreases and decreases as the thickness of the UCZ increases. The ideal thickness of UCZ is 0.3m, NCZ is 1.0m, and LCZ is 0.7m.
- The pond extracts the greatest heat possible at 110W/m^2^, with the fine sand dry at 62.48°C.
- When comparing the two cities using measured data and PMD, the solar pond is preferred in Lahore owing to the city’s superior climatic circumstances.
- Additionally, this study demonstrates that energy needs may be fulfilled effectively via the solar pond method. With an investment in Solar Pond, the industry may achieve independence from regulatory-mandated industrial gas power outages and significantly reduce their energy bills with a short payback time.

**Table:** Abbreviatios

**Symbol**	**Quantity**	**Unit**
C_p_	Specific Heat Capacity	J/kg °C
C_pw_	Specific Heat of water	J/kg °C
e_sw_	Saturated vapor pressure on the water surface	Pa
e_a_	Vapour pressure in the air	Pa
e_sat_	The saturated vapor pressure in the air	Pa
G_o_	Short wave radiation	W/m^2^
G	Incident radiation flux at solar pond surface	W/m^2^
h	Altitude	m
h_1_	Convective heat transfer coefficient between pond and ground beneath	W/m^2^/°C
h_2_	Convective heat transfer coefficient between ground and groundwater sink	W/m^2^/°C
h_c_	Convective heat transfer coefficient	W/m^2 o^C
k	Thermal conductivity	W/m°C
k_g_	Thermal conductivity of the ground	W/m°C
q_c_	Heat transfer by convection	W/m^2^
P_atm_	Pressure	Pa
q_cond_	Heat transfer by conduction	W/m^2^
q_e_	Heat transfer by evaporation	W/m^2^
q_ext_	Extraction of heat from the pond	W/m^2^
q_forced_	Forced convection heat flux	W/m^2^
q_free_	Free convection heat flux	W/m^2^
q_g_	Loss of heat to the ground	W/m^2^
q_l_	Total longwave radiation heat	W/m^2^
q_la_	Longwave radiation from the air to the pond	W/m^2^
q_lw_	Longwave radiation from the pond to the atmosphere	W/m^2^
q_r_	Heat transfer by radiations	W/m^2^
q_w_	Loss of heat to the walls	W/m^2^
S	Salinity	kg/m^3^
T	Temperature	°C
T_av_	The virtual temperature of the air	°C
t	Time	s
T_wv_	The virtual temperature of the water	°C
Δt	Timestep	s
U_g_	Overall heat transfer coefficient	W/m^2 o^C
U_2_	Speed of wind at 2m from the surface of solar pond	m/s
x	Depth of pond	m
x_g_	Distance between the perimeter of the solar pond and the position of known temperature	m
*ρ*	Density of salt	kg/m^3^
*ρ*_*g*_	Ground density	Kg/m^3^
*ρ*_*gd*_	Dry density	Kg/m^3^
