# Correction: Design and performance analysis of salinity gradient solar pond under different climatic and soil conditions

**DOI:** 10.1371/journal.pone.0315616

**Published:** 2024-12-06

**Authors:** The PLOS ONE Staff

The Funding statement for this paper is incorrect. The correct Funding statement is as follows: The authors extend their appreciation to the Deputyship for Research & Innovation, Ministry of Education in Saudi Arabia for funding this research work through the project number: IFP22UQU4310108DSR188. The publisher apologizes for the error.
